# Independent evolution of neurotoxin and flagellar genetic loci in proteolytic *Clostridium botulinum*

**DOI:** 10.1186/1471-2164-10-115

**Published:** 2009-03-19

**Authors:** Andrew T Carter, Catherine J Paul, David R Mason, Susan M Twine, Mark J Alston, Susan M Logan, John W Austin, Michael W Peck

**Affiliations:** 1 Institute of Food Research, Norwich, UK; 2 Bureau of Microbial Hazards, HPFB, Health Canada, Ottawa, Canada; 3 NRC Institute for Biological Sciences, Ottawa, Canada; 4 Centre for Chemistry and Chemical Engineering, Lund University, Lund, Sweden

## Abstract

**Background:**

Proteolytic *Clostridium botulinum *is the causative agent of botulism, a severe neuroparalytic illness. Given the severity of botulism, surprisingly little is known of the population structure, biology, phylogeny or evolution of *C. botulinum*. The recent determination of the genome sequence of *C. botulinum *has allowed comparative genomic indexing using a DNA microarray.

**Results:**

Whole genome microarray analysis revealed that 63% of the coding sequences (CDSs) present in reference strain ATCC 3502 were common to all 61 widely-representative strains of proteolytic *C. botulinum *and the closely related *C. sporogenes *tested. This indicates a relatively stable genome. There was, however, evidence for recombination and genetic exchange, in particular within the neurotoxin gene and cluster (including transfer of neurotoxin genes to *C. sporogenes*), and the flagellar glycosylation island (FGI). These two loci appear to have evolved independently from each other, and from the remainder of the genetic complement. A number of strains were atypical; for example, while 10 out of 14 strains that formed type A1 toxin gave almost identical profiles in whole genome, neurotoxin cluster and FGI analyses, the other four strains showed divergent properties. Furthermore, a new neurotoxin sub-type (A5) has been discovered in strains from heroin-associated wound botulism cases. For the first time, differences in glycosylation profiles of the flagella could be linked to differences in the gene content of the FGI.

**Conclusion:**

Proteolytic *C. botulinum *has a stable genome backbone containing specific regions of genetic heterogeneity. These include the neurotoxin gene cluster and the FGI, each having evolved independently of each other and the remainder of the genetic complement. Analysis of these genetic components provides a high degree of discrimination of strains of proteolytic *C. botulinum*, and is suitable for clinical and forensic investigations of botulism outbreaks.

## Background

The species *Clostridium botulinum *consists of a group of four physiologically and phylogenetically distinct Gram-positive obligately anaerobic bacteria that share the common feature of producing the highly potent botulinum neurotoxin [1]. Organisms belonging to two of these groups are associated with the majority of cases of human botulism. *C. botulinum *Group I (proteolytic *C. botulinum*) is a mesophilic organism that is responsible for foodborne botulism, wound botulism, adult intestinal botulism and infant botulism. *C. sporogenes *is considered to be a non-toxigenic version of proteolytic *C. botulinum *[2]. *C. botulinum *Group II (non-proteolytic *C. botulinum*) is a psychrotrophic organism associated with most cases of foodborne botulism not attributed to Group I [3,4]. The botulinum neurotoxins are the most potent toxins known, with as little as 30–100 ng constituting a potentially fatal dose [5], and are considered to be a bioterrorism threat [6].

Seven major types of botulinum neurotoxin (types A to G), and a significant number of sub-types have been described. For example, four sub-types of type A toxin (termed A1, A2, A3, A4) have been identified [7-9]. Sub-types are defined as differing by at least 2.6% at the amino acid level [7,10]. Proteolytic *C. botulinum *strains form neurotoxin of types A, B, or F, and dual-toxin forming strains have also been described [2]. Additionally, some strains possess two neurotoxin genes, but only form one active neurotoxin. For example, A(B) strains possess a type A and type B neurotoxin gene, but only form type A neurotoxin. Non-proteolytic *C. botulinum *strains form a single neurotoxin of types B, E, or F. Each neurotoxin protein comprises a light chain and heavy chain. The light chains possess endopeptidase activity and cleave proteins in the SNARE complex leading to flaccid muscle paralysis, and potentially respiratory failure [11].

The neurotoxin genes are associated with other genes within the neurotoxin cluster, and two major cluster types are recognised. The most studied neurotoxin cluster in proteolytic *C. botulinum *is termed the ha plus/orf-X minus cluster. It is commonly associated with type A1 and type B neurotoxin genes [9,12,13], and is present in the genome of the sequenced type A1 strain ATCC 3502 used as a hybridisation reference in this work [14]. This cluster comprises genes for the neurotoxin (*cntA*), three haemagglutinins (HA) (*cntC, cntD, cntE*), non-toxic-non-haemagglutinin (NTNH) (*cntB*), and a positive regulatory protein (*cntR*). The second cluster type is called the ha minus/orf-X plus cluster. In the case of proteolytic *C. botulinum*, this cluster is most frequently associated with type A2, A3, A4 and F toxin genes, and the type A1 gene in A(B) strains [9,12,13]. This cluster includes genes for the neurotoxin, NTNH and CntR (historically also known as p21 [9,13]), lacks the three genes encoding HA, and additionally contains a group of three open reading frames (*orf-X1*, *orf-X2*, *orf-X3*) and a single CDS (coding sequence) (*p47*) all of unknown function.

The genome sequence of proteolytic *C. botulinum *strain ATCC 3502 (NCTC 13319, Hall 174) has been recently completed, and consists of a chromosome (3.9 Mbp) and plasmid (16.3 kbp), which contain 3,650 and 19 coding sequences (CDSs), respectively [14]. A DNA microarray was designed based on this sequence, and initial tests revealed that two prophages and a plasmid present in the genome of strain ATCC 3502 were absent from 11 test strains of proteolytic *C. botulinum *and *C. sporogenes*, and that the DNA microarray could be used to discriminate between strains of proteolytic *C. botulinum *[14]. The 11 test strains shared a minimum of 84% of the CDSs of ATCC 3502, but were significantly diverged from other sequenced clostridial species, demonstrating the wide phylogenetic distance between different clostridia [14].

The flagellar glycosylation island (FGI) also showed evidence of diversity between strains of proteolytic *C. botulinum *[14]. The ATCC 3502 genome contains a large putative FGI comprising CDSs CBO2666-2729. These are flanked by the CDSs for flagellar structural proteins FlgB (CBO2665), FliD and the flagellin structural subunits FlaA1 (CBO2730) and FlaA2 (CBO2731). The FGI can be divided into two distinct regions [14]. CBO2678-2689 are CDSs similar to those involved in capsular polysaccharide biosynthesis in Group B *Streptococcus *(designated FGI-I, flanked by putative flagellin structural genes CBO 2666 and CBO 2695), whereas CBO2696-2729 represent CDSs with sequence similarity to those involved in the modification of *Campylobacter jejuni *flagellins with nonulosonic acids (designated FGI-II, CBO2696-CBO2729) [14].

In order to extend our understanding of phylogenetic relationships and the biology of proteolytic *C. botulinum*, an extensive comparative genomic indexing study has been carried out involving 58 strains of proteolytic *C. botulinum*, 2 strains of non-proteolytic *C. botulinum*, and 3 strains of *C. sporogenes *using DNA microarrays based on the genome sequence of strain ATCC 3502. We have assessed the evolution of the neurotoxin gene and cluster and flagellar glycosylation island (FGI) in relation to the remainder of the genetic complement. We have also identified important links between CDSs contained within the FGI and sugars associated with post-translational modification of flagella, and discovered a new neurotoxin A sub-type associated with UK wound botulism cases.

## Methods

### Bacterial strains and preparation of DNA

*C. botulinum *and *C. sporogenes *strains used in this work, together with the type of neurotoxin formed, their origin, source and date of isolation are listed in Table 1. Before use, all strains were checked for purity (consistent colony morphology) and lack of contamination by growth on PYGS plates under both aerobic and anaerobic atmospheres [15]. Proteolytic activity was determined by growth on Reinforced Clostridial Medium (RCM) containing 5% (w/v) skim milk [16] and lipase activity on McClung Toabe egg yolk medium [17]. Strains were also checked for presence of type A, B and F neurotoxin genes by PCR using 100 ng genomic DNA as template with primer pairs NKB-1 (5'-GATACATTTACAAATCCTGAAGGAGA-3') and NKB-5 (5'-AACCGTTTAACACCATAAGGGATCATAGAA-3') which generate a 2278 bp PCR product for the type A neurotoxin gene; B-1A (5'-GATGGAACCATTTGCTAG-3') and B2-D (5'-AACATCAATACATATTCCTGG-3') which generate a 1284 bp PCR product for the type B neurotoxin gene [18]; and BONTFF2 (5'-GTGCTTATTATGATCCTAATTATTTAACC-3') and BONTFR2 (5'-CCATACTTCCATTGAAAATAATCTTTATA-3') which, using the same reaction conditions, give a 765 bp PCR product for the type F neurotoxin gene (data not shown). The type(s) of neurotoxins formed by each strain was established by sero-neutralisation and the mouse bioassay [19,20].

**Table 1:** Summary of details of *Clostridium *strains tested in microarray.

Bacterium and toxin type	Strain	Origin	Location (year)	source
Proteolytic *C. botulinum*				
A1	ATCC 3502	Foodborne botulism? (canned peas)	California, USA (early 1920s)	IFR
A1	NCTC 3805	Environment (bovine)	Nevada, USA (1919)	IFR
A1	VL1	Environment (ham)	Netherlands (1970s)	IFR
A1	16037	Foodborne botulism (canned tomato juice)	Idaho, USA (1974)	IFR
A1	ATCC 25763	Foodborne botulism/environment?	unknown	IFR
A1	96A	Foodborne botulism/environment?	unknown	IFR
A1	CDC 1690	Foodborne botulism (home canned spaghetti)	Tennessee, USA (1977)	IFR
A1	NCTC 7272	Environment?	(before 1947)	IFR
A1	17A	Foodborne botulism/environment?	unknown (1963)	HC
A1	F9801A	Infant botulism (faeces)	Quebec, Canada (1998)	HC
A1	FE0101AJO	Foodborne botulism (faeces)	Quebec, Canada (2001)	HC
A1	FE0205A1AK	Infant botulism (faeces)	Alberta, Canada (2002)	HC
A1	F9604A	Infant botulism (faeces)	Alberta, Canada (1996)	HC
A1	MUL0109ASA	Environment (mullet fish)	Gulf of Kuwait, Kuwait (2001)	HC
A2	NCTC 9837	Foodborne botulism (canned fish)	London, UK (1955)	IFR
A2	ZK3	Environment (ground nuts)	Bedford, UK (1950s)	IFR
A3	NCTC 2012	Foodborne botulism (wild duck paste)	Loch Maree, UK (1922)	IFR
				
A1(B)	NCTC 11199	Infant botulism (faeces)	Southend, UK (1978)	IFR
A1(B)	CDC 5001	Foodborne botulism (sautéed onions)	Illinois, USA (1983)	IFR
A1(B)	CDC 13280	Foodborne botulism (home canned peppers)	Colorado, USA (1972)	IFR
A1(B)	MDa10	Infant botulism (faeces)	USA (1980s)	IFR
A1(B)	NCTC 2916	Foodborne botulism (canned corn)	Colorado, USA (1929)	IFR
A1(B)	FE0207AMB	Foodborne botulism (faeces)	Ontario, Canada (2002)	HC
A1(B)	FE0303A1YO	Infant botulism (faeces)	Ontario, Canada (2003)	HC
A1(B)	FE9504ACG	Infant botulism (faeces)	Quebec, Canada (1995)	HC
A1(B)	FE9909ACS	Infant botulism (faeces)	Alberta, Canada (1999)	HC
A1(B)	PE0101AJO	Foodborne botulism (pork cutlets)	Quebec, Canada (2001)	HC
A1(B)	FE0602ALS	Infant botulism (faeces)	Alberta, Canada (2006)	HC
A1(B)	CJ0611A	Foodborne botulism (carrot juice)	Ontario, Canada (2006)	HC
A1b	CDC 588	Foodborne botulism (home canned carrots)	Ohio, USA (1976)	IFR
Ba4	CDC 657	Infant botulism (faeces)	Texas, USA (1976)	IFR
A5(B)	H0 4244 0055	Wound botulism (pus)	Sheffield, UK (2004)	IFR
A5(B)	H0 4402 065	Wound botulism (pus)	Dudley, UK (2004)	IFR
A5(B)	H0 4464 107	Wound botulism (wound)	Manchester, UK (2004)	IFR
A5(B)	H0 4068 0341	Wound botulism (wound)	London, UK (2004)	IFR
				
B	NCTC 7273	Foodborne botulism? (beans?)	UK? (before 1947)	IFR
B	2345	Foodborne botulism/environment?	(before 1970s)	IFR
B	NCTC 3815	Environment? (cheese)?	USA (1930s)	IFR
B	BL81/25	Foodborne botulism/environment?	unknown	IFR
B	213B	Foodborne botulism/environment?	unknown	IFR
B	BL 143	Environment (fish)	Bedford, UK (1969)	IFR
B	BL 150	Environment (fish)	Bedford, UK (1970)	IFR
B	CDC 15044	Foodborne botulism (home canned blackberries)	Kentucky, USA (1973)	IFR
B	NCIB 4301	Foodborne botulism/environment?	unknown	IFR
B	CDC 7827	Infant botulism (faeces)	Nevada, USA (1991)	IFR
B	IB1-B	Infant botulism (faeces)	Ontario, Canada (1979)	HC
B	368B	Infant botulism (faeces)	California, USA (1976)	HC
B	920A276	Infant botulism (faeces)	California, USA (1976)	HC
B	FE0507BLP	Infant botulism (faeces)	Ontario, Canada (2005)	HC
B	13441-77	Infant botulism (faeces)	California, USA (1977)	HC
B	MRB	Foodborne botulism (mushrooms)	Quebec, Canada (1973)	HC
				
Bf	CDC 3281	Infant botulism (faeces)	New Mexico, USA (1980)	IFR
Bf	FE9508BRB	Foodborne botulism (faeces)	Quebec, Canada (1995)	HC
Bf	FE9508BPD	Foodborne botulism (faeces)	Quebec, Canada (1995)	HC
Bf	PA9508B	Foodborne botulism (pâté)	Quebec, Canada (1995)	HC
				
F	Langeland	Foodborne botulism (liver paste)	Langeland, Denmark (1960)	HC
F	Walls 8G	Environment (crab)	Virginia, USA (1968)	IFR
F	H461297F	Environment (honey)	Wisconsin, USA (1998)	HC
*C. sporogenes*				
	NCIMB 10696	Environment (soil)	USA (1920s?)	IFR
	NCDO 1792	Environment (aseptic cheese)	Reading, UK? (1960s?)	IFR
	NCDO 1710	Gas gangrene	London, UK (1920)	IFR
Non-proteolytic *C. botulinum*				
E	GA9709EHS	Foodborne botulism (gastric liquid)	Quebec, Canada (1997)	HC
E	HNB0804E	Environment (honey)	New Brunswick, Canada (2004)	HC

Source: IFR = IFR culture collection; HC = Health Canada culture collection.

Genomic DNA was purified from exponentially growing cells, digested with Sau3A1 and labelled with fluorescent nucleotides as previously described [14] except that Cy5- or Cy3-dUTP (GE Healthcare, UK) was substituted for Cy5- or Cy3-dCTP. The isolation of plasmid DNA followed the method outlined by O'Sullivan and Klaenhammer [21]. For restriction enzyme analysis, the manufacturer protocols (New England BioLabs, USA) were followed with the addition of spermidine to a final concentration of 4 mM. Digests were analyzed by standard gel electrophoresis in 1.5% agarose.

### Construction of the *C. botulinum *ATCC 3502 DNA microarray

The microarray [14] included probes for 3433 genomic CDS, for 19 CDSs of plasmid pBOT3502, and for neurotoxin-associated genes absent in strain ATCC 3502. These included probes for CDSs of the gene clusters commonly associated with neurotoxins type A2, A3, A4 and F (*i.e. p47*, *orf-X1*, *orf-X2*, *orf-X3 *and *lycA*); for *cntR *of type F; and two probes each (N- and C-terminus probes) for *cntA *(neurotoxin CDS) of types A, B, and F. Probes were designed using GenBank database sequences (available in July 2005), and PCR amplified using DNA from strains NCTC 9837 (*p47*, *orf-X1*, *orf-X2*, *orf-X3 *and *lycA*), Langeland (*cntR/F, cntA/F-N *and *cntA/F-C*), ATCC 3502 (*cntA/A-N *and *cntA/A-C*), and NCTC 3815 (*cntA/B-N *and *cntA/B-C*). The microarray probe to the haemagglutinin gene *cntC *(CBO0803; previously *ha34*) failed to give a satisfactory signal and these microarray data were excluded from the analysis. The overall extent of genome coverage is similar to that achieved previously for other DNA microarrays [22-24]. GenBank accession numbers for genomes used in this work are: A1 strain ATCC 3502, AM412317; ATCC 3502 plasmid pBOT3502, AM412318; A1 strain ATCC 19397, CP000726; A3 strain Loch Maree, CP000962; F strain Langeland, CP000728; A1(B) strain NCTC 2916, NZ_ABDO00000000 (genome in progress); strain Bf, NZ_ABDP00000000 (genome in progress); *C. sporogenes *strain ATCC 15579, NZ_ABKW00000000 (genome in progress). The microarray design has been deposited with ArrayExpress (accession number A-MEXP-791).

### Microarray hybridisation and data analysis

Each experiment combined 2 μg Cy5-dUTP-labelled ATCC 3502 (reference) DNA and 2 μg Cy3-dUTP-labelled test DNA, and was performed on a minimum of four probe set replicates as described previously [14]. DNA microarrays were scanned using an Axon GenePix 4000B microarray laser scanner (Axon Instruments, CA, USA). The data from detected features was initially processed using the GenePix Pro v.6.0 software supplied with the scanner.

The R package arrayMagic v.1.14.0 [25] was used to assess the quality of the hybridisations by generating a diagnostic plot showing the pairwise similarities between all hybridisations. The pairwise similarity score (S_*ab*_) was calculated by arrayMagic via S_*ab *_= MAD_*i*_(X_*ia *_- X_*ib*_), where for each pair of arrays (*a *and *b*) X_*ia *_is the log-ratio of the *i*-th probe on the *a*-th array, and the MAD (median absolute deviation) is taken over all CDSs. The hierarchical clustering diagram generated used the similarity scores as a measure of the 'distance' between arrays. In this way the fidelity of the microarray technical replicates could be assessed (arrayMagic's R script, experiment description file and diagnostic plot are available on request). The data for replicates that did not group together were discarded and the hybridisation experiments repeated with a fresh preparation of genomic DNA. Array data were further analysed using the GeneSpring GX package (Agilent Technologies) using Lowess normalisation. In order to correct for uneven printing or for probes which routinely gave a high or low signal, data were further normalised by using as a control hybridisation data from ATCC 3502 × ATCC 3502 dye-swap experiments (four microarrays) on a per CDS basis.

Pearson Correlation coefficients were calculated for the normalised signal ratios associated with probes for all chromosomal and pBOT3502 CDSs and used to create a similarity matrix for all 61 strains of proteolytic *C. botulinum *and *C. sporogenes*. The similarity matrix was subjected to the average linkage clustering method using GeneSpring GX software.

The data generated by probes for neurotoxin cluster genes not found in ATCC 3502 were processed separately as there was no competing reference DNA during hybridisation. Whereas a signal channel ratio of 0.55 was taken as the cut-off between a presence or absence of hybridisation for chromosomal genes, a ratio greater than 5.0 was taken as a positive hybridisation for CDSs not in ATCC 3502. This gave results that agreed well with known genome sequences of *C. botulinum *in the GenBank database. Data for probes to the ATCC 3502 neurotoxin gene cluster itself used a cut-off point of 0.40 to compensate for the fact that all hybridisations had been performed using ATCC 3502 DNA as the reference material.

### Validation of microarray

**Figure 1:**
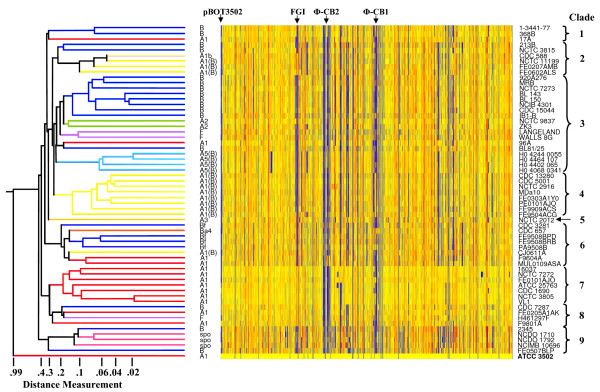
**Whole genome analysis of 61 strains of proteolytic *C. botulinum *and *C. sporogenes***. Each row of the heatmap represents a strain (indicated at right), and its branch on the dendrogram is coloured according to type of neurotoxin formed (indicated at left of heatmap; spo refers to *C. sporogenes*). Although lost at this resolution, each microarray probe is represented by a vertical column within this row, from left to right first the 19 probes for each CDS of ATCC 3502 plasmid pBOT3502, followed by probes for chromosomal CDSs, from CBO3648 to CBO001. The colour of each column in the heatmap is an indicator of test signal over reference (ATCC 3502) signal channel ratio. Yellow columns represent probes which hybridised to both test and reference isolates equally, those in blue hybridised more strongly to the reference strain, and those in red hybridised more strongly to the test strain. Microarray features with fluorescent signals lower than 100 units (background noise), plus those CDSs not represented on the microarray are coloured grey. Distance measurements between 0 and 1.0 are indicated in the non-linear scale underneath the dendrogram. Clades 1 to 9 (brackets at right), are groups of strains which cluster at a distance measurement value of 0.3. The four main regions of variability (clusters of blue-coloured columns) are CDSs associated with pBOT3502, the Flagellar Glycosylation Island (FGI), and the two prophages, Φ-CB1 and Φ-CB2 (indicated above heatmap).

**Figure 2:**
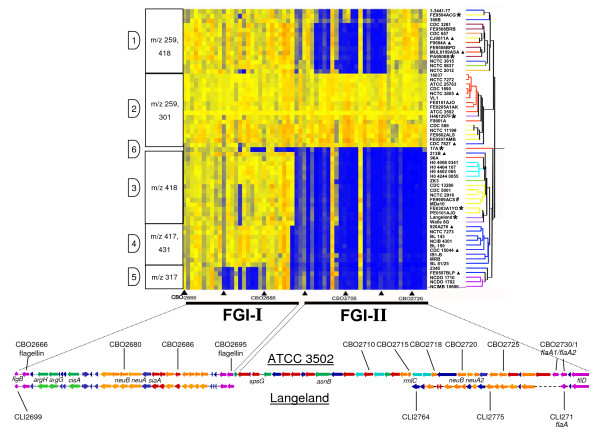
**Heatmap comparison of CDSs in the Flagellar Glycosylation Island (FGI)**. Top: See Figure 1 for explanation of heatmap format. Strains are ordered by FGI CDS data; dendrogram and strain names at right are coloured (as for Figure 1) for type of neurotoxin formed. Filled triangles (bottom of heatmap): approximately 10 CDS intervals (data for some CDSs are absent). CDSs of FGI-I (CBO2666 – CBO2692) are highly conserved and those of FGI-II (CBO2696 – CBO2729) are less so. Hybridisation profiles divided strains into 6 divisions, numbered at left. Mass of glycans detected by mass spectrometry analysis of FlaA proteins are symbolized in boxes at left. Strains examined by top down mass spectrometry are marked with a filled triangle. Top down profiles of flagellin from strains marked with a single asterisk and the complete structure of the posttranslational modification for strain FE9909ACS (hatch symbol) have been combined with previously published data [31]. Bottom: FGI sequence comparison of proteolytic *C. botulinum *strains ATCC 3502 (FGI division 2), top, and Langeland type F (FGI division 3), below, confirms heatmap data. Synteny within FGI-I region (left) contrasts markedly with FGI-II; here ATCC 3502 contains approximately 20 CDSs not found in Langeland and displays less synteny and homology with the CDSs of the Langeland FGI-II. Heatmap data show that Langeland FGI contains many of the genes found in FGI-I of ATCC 3502 (yellow columns) while is still missing a large number of genes found in FGI-II of ATCC 3502 (blue columns).

**Figure 3:**
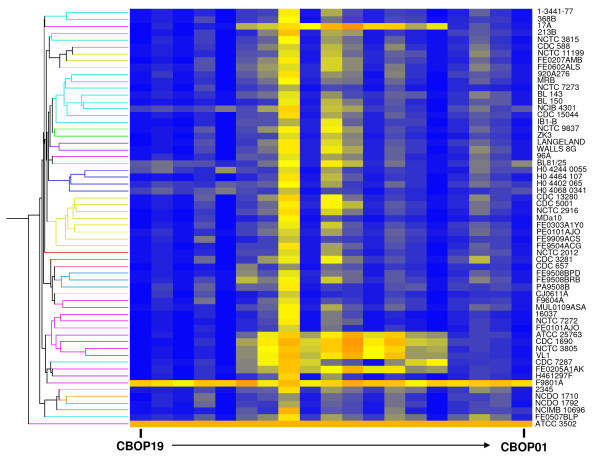
**Plasmid pBOT3502 of strain ATCC 3502 shares CDSs with other strains of proteolytic *C. botulinum***. Magnification of the first 19 columns (probes for pBOT3502 CDSs) of the heatmap presented in Figure 1. Yellow/orange bars depict CDSs with significant homology to the probe; only strain F9801A possesses all 19 CDSs (CBOP1–CBOP19), suggesting it carries a plasmid closely related to pBOT3502.

**Figure 4:**
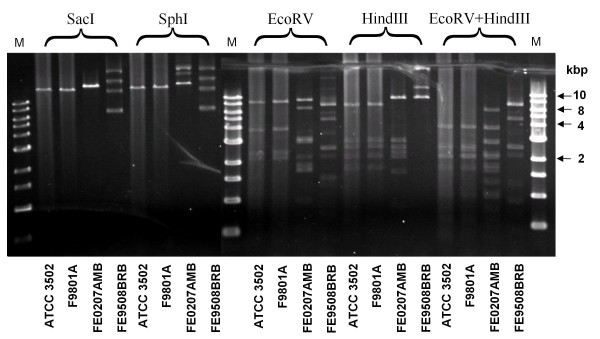
**Restriction digests of plasmids carried by four strains of proteolytic *C. botulinum***. Ethidium bromide-stained agarose gel photographed under UV light showing plasmid DNA extracted from four strains (indicated at bottom) following digestion with different restriction endonucleases (indicated at top). The plasmids carried by strains ATCC 3502 and by F9801A are clearly closely related or identical (see Figure 3). Lanes containing size markers (up to 10 kilobase pairs, kbp) are labelled M.

The microarray data were validated for biological significance using CDSs within the clostridial flagellar glycosylation island (FGI) and plasmid pBOT3502. The DNA sequence of 28 CDSs from the FGI-I (Figures 1 and 2) of ATCC 3502 and proteolytic *C. botulinum *type F strain Langeland, matched by annotation using genomic context and BlastP, was compared to the signals observed by microarray analysis. The highest sequence similarity between two homologous CDSs (CBO2682 and CLI_2747) corresponding to an absence of microarray hybridisation was 84.8%. The lowest sequence similarity between two homologous CDSs (CBO2683 and CLI_2748) that hybridised to the microarray was 85.7%, giving a minimum value of approximately 85% sequence identity between CDSs for a positive microarray hybridisation result. A similar percentage was previously reported for studies with *Candida *[26] and *Helicobacter pylori *[27]. Further validation was carried out by analysis of data for the 19 probes to the plasmid pBOT3502. Using a cut-off value of 0.30 (because of very high signals), only one strain, F9801A, gave a positive microarray signal for all 19 CDSs (Figure 3). Subsequent tests demonstrated that this strain, but not two others that were tested, contained a plasmid that shared identical restriction sites to that of pBOT3502 (Figure 4). Additionally, pBOT3502 contains five CDSs (CBOP15–CBOP19) that are dedicated to the synthesis and secretion of the bacteriocin, boticin [14]. However apart from F9801A, no other strain gave a microarray signal for these probes, including *C. botulinum *strain 213B. This strain carries a plasmid bearing the genes for boticin B [28], so might have been expected to give a positive signal. However, alignment of the 1 kb sequence from strain 213B with that of pBOT3502 showed that sequence identity over this region, spanning pBOT3502 CDSs CBOP16 and CBOP17, was only 52.1% which would fail to give a positive microarray signal.

### Isolation and mass spectrometry analysis of flagellin proteins

Flagellin proteins were isolated [29] and mass spectrometry studies of intact flagellin proteins were carried out as described in earlier studies [30,31]. In some cases a large precipitate was observed in dialysed flagellin preparations. Protein isolates were evaporated to dryness in a Savant SpeedVac (Thermo Fisher Scientific UK) before resuspending in 5–10 μl of formic acid. The sample was agitated gently to dissolve protein and diluted 10-fold with hexafluoroisopropanol. Samples were infused into a hybrid quadrupole time-of-flight mass spectrometer (Micromass Q-TOF2, Waters Corporation, MA, USA) at a flow rate of 0.5–1.0 μl/min [30,31]. Top down mass spectrometry experiments were performed as described by [30], using argon collision gas with collision energies ranging from 20–30V.

### Sequencing of sub-type A5 neurotoxin genes carried by wound botulism strains

To lower the risk of PCR-based errors, the A5 genes were sequenced using non-cloned PCR products. Initial 3.8 kb PCR products of the majority of the gene CDS were generated using a LongRange PCR kit (Qiagen, UK), with primers BONTAF1 (5'-GCAACCAGTAAAAGCTTTTAAAATTC-3'), BONTAR1 (5'-CCATCCATCATCTACAGGAATAAA-3') and 100 ng genomic DNA as template. PCR products were purified using DyeEx 2.0 spin columns (Qiagen). Sequencing was carried out using an AbiPrism 3730 capillary sequencer. Sequence of the entire 3.8 kb PCR product was achieved by designing primers using available sequence data and by 'walking' forward on both strands. Comparison of the 3.8 kb sequence with published examples of *C. botulinum *neurotoxin genes showed that the A5 neurotoxin sub-type was a close relative of the A1 sub-type, which implied a similar neurotoxin locus structure. Therefore to amplify DNA containing the 5' and 3' ends of the A5 neurotoxin genes a series of PCR primers were designed that would recognise the *cntB *gene (encoding NTNH) and the transposase that flank the A1 neurotoxin gene of ATCC 3502 (CBO0805 and CBO0807 respectively). PCR was performed using 'outward facing' primers recognising the 3.8 kb sequence combined with these two sets of primers. PCR products that were of the expected size were sequenced. All sequencing fragments were assembled using the ContigExpress programme of the Vector NTI Advance 10 software package (Invitrogen). Comparison of the completed A5 neurotoxin gene sequence with that of published examples of other *C. botulinum *neurotoxin genes together with phylogenetic tree construction was carried out using the AlignX programme of this package. The A5 neurotoxin gene of four strains associated with UK cases of wound botulism was sequenced and found to be identical.

### Accession Numbers

A representative of the sub-type A5 neurotoxin gene sequence from wound botulism strain H0 4402 065 was deposited in GenBank (accession number EU679004). Microarray data have been deposited with Array Express (accession number E-MEXP-1637).

## Results and discussion

### Whole genome analysis

The 61 strains of proteolytic *C. botulinum *and *C. sporogenes *tested in the present study were selected to represent diverse origins. They had originally been isolated at different times over a period of more than 80 years, from the environment (17 strains (including unknowns)) or associated with various forms of botulism (foodborne (20 strains), infant (17 strains), and wound (4 strains)). The strains were of different toxin types; type A toxin gene (17 strains), type B toxin gene (16 strains), type F toxin gene (3 strains), dual toxin genes (22 strains), and no toxin gene (3 strains of *C. sporogenes*) (Table 1). The CDS content of the 61 test strains was indexed in relation to the genome of proteolytic *C. botulinum *strain ATCC 3502 (Figure 1). The dendrogram and heatmap (Figure 1) were derived from this whole genome analysis, and show that all the strains of proteolytic *C. botulinum *and *C. sporogenes *share a high degree of genetic relatedness (e.g. clustering distance or branch-lengths in the dendrogram were short with a high proportion of shared CDSs in the heatmap (coloured yellow)). Most major branch points in the dendrogram occurred at distance measurements of between 0.20 and 0.44. A distance measurement value of 0.30 separated the 61 strains of proteolytic *C. botulinum *and *C. sporogenes *into nine clades (excluding ATCC 3502) (Figure 1). The strains did not group together according to the location, environment, time of isolation, or the type of botulism with which they were associated (Figure 1). This lack of grouping probably reflects the wide range of sources of the strains, and has been reported previously by workers using other typing methods [8,32]. The predominance of yellow shading in the heatmap indicates that the 11 strains in clades 7 and 8 (Figure 1) were most closely related to the reference strain (ATCC 3502). For example, they shared the same FGI. While nine of fourteen type A1 neurotoxin strains (as ATCC 3502) were present in these two clades, a type B and type F strain were also present. Indeed, most clades contained strains of more than one toxin type (or sub-type), and most toxin types (or sub-types) were represented in more than one clade, suggesting that the evolution of the neurotoxin genes has not paralleled that of the remainder of the genetic complement. For example, clade 3 contains nine type B strains, one type A1 strain, two type A2 strains, two type F and four type A5(B) strains (the novel type A5(B) strains are described below), and clade 9 contained two type B strains and three *C. sporogenes *strains (Figure 1), confirming the close relationship between proteolytic *C. botulinum *and *C. sporogenes *(e.g. [2,8,14]). Two clades, however, contained strains of just one toxin type. Clade 4 contained eight closely-related North American-isolated type A1(B) strains, and clade 7 comprised seven closely-related type A1 strains (Figure 1). Clade 5 contained a single strain (NCTC 2012, Loch Maree) that forms type A3 toxin. Interestingly, other genomic indexing methods (MLST, AFLP, VNTR) also found this strain to be unique and well separated from other strains of proteolytic *C. botulinum *[8,33,34]. In addition to the nine clades identified, further sub-groupings were identified within each clade, often of the same toxin type (or sub-type). Some strains appeared highly similar to each other when compared to the genome of ATCC 3502. These strains included the three type Bf strains isolated from two patients and food following a foodborne botulism outbreak in Quebec that grouped closely together within clade 6, and the four strains associated with wound botulism in the UK within clade 3 (Figure 1). Most of the differences in microarray data between the three type Bf strains were distributed around the signal channel ratio cut-off point of 0.55, suggesting that these apparent differences may reflect background noise associated with this type of analysis. Indeed, it is likely that these three Bf strains are identical, as they were isolated from a pâté and clinical samples from the same outbreak. On the other hand, the wound botulism strains showed some clear differences in their genetic content. Other genomic indexing methods (e.g. MLST, PFGE, AFLP, VNTR) have given a broadly similar pattern to that found in the present study, with groups of small numbers of closely-related strains generally of the same toxin type grouping together, with several distinct groups for each toxin type [8,32-34]. There are, however, a number of interesting anomalies that might be interpreted as evidence for horizontally acquired genetic information, and therefore worthy of further study, for example the type B strain 2345 that groups most closely with the *C. sporogenes *strains.

**Figure 5:**
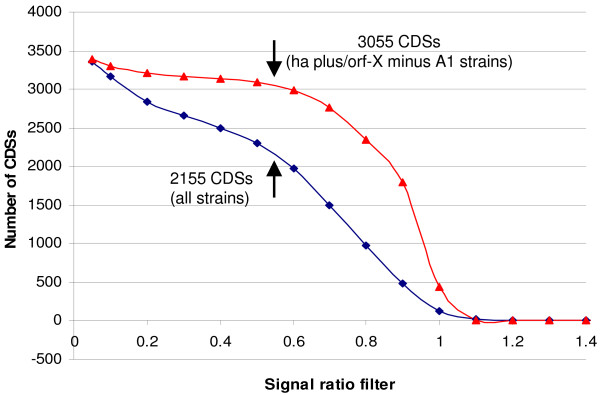
**Core set of CDSs of proteolytic *C. botulinum/C. sporogenes***. Microarray data were filtered to calculate numbers of CDSs which were shared by all strains at a given signal channel ratio. A cut-off value of 0.55 (arrows) was chosen as most appropriate to exclude CDSs that are absent or diverged from their ATCC 3502 counterparts. From the plots presented here this ratio value indicates a core set of 2155 CDSs that are shared by all 61 strains tested (filled diamonds), and 3055 CDSs that are shared by all 10 *C. botulinum *ha plus/orf-X minus A1 strains in clades 7 and 8 (filled triangles).

It is estimated that the core gene set for all 61 strains of proteolytic *C. botulinum *and *C. sporogenes *tested was 2155 CDSs (Figure 5). This is approximately 63% of the CDSs of ATCC 3502 represented by probes on the microarray, and considerably higher than the value of 20% previously reported for 75 strains of *C. difficile *[35]. This further confirms the close relationship of proteolytic *C. botulinum *and *C. sporogenes *and indicates that exchange of genetic information with other species has occurred less frequently than in *C. difficile*. Apart from the neurotoxin gene cluster itself, which although significant in terms of biological impact, represents a very small part of the genome, four main areas of divergence were identified; the plasmid (pBOT3502), the flagellar glycosylation island (FGI) and the two prophages (Figure 1). Together these account for approximately 4.6% of the DNA (plasmid plus chromosome) of ATCC 3502. It was previously estimated that two type A1 strains shared 95–96% of their CDSs with ATCC 3502 [14], but the two strains included in this previous study are now revealed to be very close relatives. Indeed, it was estimated that the core gene set for the ten closely-related type A1 strains in clades 7 and 8 was 3055 CDSs, equating to 89% of the CDSs of ATCC 3502 (Figure 5).

Additionally, two strains of non-proteolytic *C. botulinum *type E were tested, but too many CDSs were either absent or highly diverged for meaningful data to be derived (data not shown). It was previously reported that a strain of non-proteolytic *C. botulinum *type B and a strain of *C. difficile *were also too divergent to give a meaningful response on this microarray [14]. The poor hybridisation of DNA from the three strains of non-proteolytic *C. botulinum *to the microarray reflects the wide evolutionary and phylogenetic distance between proteolytic *C. botulinum *and non-proteolytic *C. botulinum*. This is a direct result of the species "*C. botulinum*" being defined not on the basis of a close evolutionary or phylogenetic relationship, but on the basis of the disease caused [3].

### Neurotoxin cluster arrangement – Single toxin gene strains

**Figure 6:**
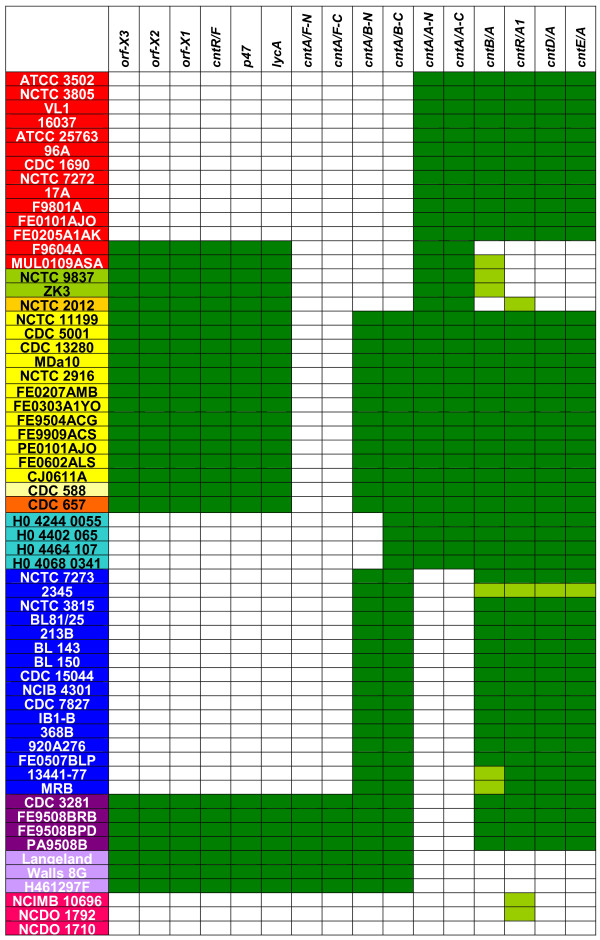
**Summary of microarray data for 16 neurotoxin gene cluster probes**. Names of proteolytic *C. botulinum *or *C. sporogenes *strains (left) are coloured according to type of neurotoxin(s) formed: red, A1; green, A2; ochre, A3; yellow, A1(B); pale yellow, A1b; orange, Ba4; pale blue, A5(B); blue, B; purple, Bf; lilac, F; magenta, *C. sporogenes*. Positive hybridisation results for microarray probes (above) are coloured green, borderline positives are pale green.

The type A1 neurotoxin gene is normally present in the ha plus/orf-X minus type cluster, while the ha minus/orf-X plus cluster is more commonly associated with type A2, A3, A4 and F neurotoxin genes [9,36,37]. Twelve of the fourteen type A1 neurotoxin strains tested contained the ha plus/orf-X minus cluster, but in two strains (F9604A and MUL0109ASA) the type A1 neurotoxin gene appears to be in a ha minus/orf-X plus cluster (Figure 6). This arrangement has also been recently reported for a small number of other type A1 strains [18,38]. In the present study, the genes (*p47*, *orf-X1*, *orf-X2*, *orf-X3 *and *lycA*) that are only present in the ha minus/orf-X plus cluster were always present together (26 strains), with no strain possessing only part of this cluster. The neurotoxin gene of the two type A2 strains (NCTC 9837 and ZK3) and one type A3 strain (NCTC 2012 – Loch Maree) was also present in a ha minus/orf-X plus cluster (Figure 6), as expected [9,13,18,37]. Although the two type A1 ha minus/orf-X plus strains (F9604A and MUL0109ASA) had the same neurotoxin cluster as the type A2 and A3 neurotoxin-forming strains (Figure 6), they were in different clades well separated from each other and from the other type A1 neurotoxin-forming strains (Figure 1). Instead these two type A1 ha minus/orf-X plus strains grouped with a type Ba strain (CDC 657) (Figure 1, clade 6). The type A neurotoxin gene in strain CDC 657 (type A4) is also in a ha minus/orf-X plus cluster [9]. Since previous studies using AFLP, MLST and MVLA have shown two other closely-related type A1 ha minus/orf-X plus strains (CDC 297 and CDC 5328) also grouped with strain CDC 657 [8,33,34,38], it is likely that the four type A1 ha minus/orf-X plus strains are closely related. Strain CDC 657 may have recently acquired a type B neurotoxin gene, or the type A1 ha minus/orf-X plus strains may have lost a type B neurotoxin gene. It is noted that the neurotoxin genes in CDC 657 are present on a plasmid, while the type A gene in at least one type A1 ha minus/orf-X plus strain (CDC 5328) is located on the chromosome [39].

The 16 type B strains gave an almost identical hybridisation pattern, with all neurotoxin genes present in a ha plus/orf-X minus cluster (Figure 6). This is consistent with previous reports [9,12]. Strain MRB had a weak signal for *cntB *(encoding NTNH), this may reflect the mosaic structure of *cntB*, and a previous genetic crossover event between two types of neurotoxin gene cluster [2,9,40]. The three type F strains gave a ha minus/orf-X plus pattern (Figure 6), an arrangement consistent with that reported in the genome sequence for strain Langeland (CDSs CLI_0845 to CLI_0850). However, while strains Langeland and Walls 8G grouped together in the whole genome analysis (Figure 1, clade 3), strain H461297F grouped with type A1 strains, providing further evidence that the neurotoxin gene clusters are not evolutionarily tied to their host organism [8,9,13].

The genes *cntR/A1 *and *cntR/F *(sometimes called *p21*) encode closely related sigma 70 factors involved in regulation of the neurotoxin genes [9,13]. The probe designed to be specific for *cntR/A1 *gave a positive result with all strains that possessed a neurotoxin gene in a ha plus/orf-X minus cluster (Figure 6). Similarly the probe designed to be specific for *cntR/F *gave a positive result with all strains that possessed a neurotoxin gene in a ha minus/orf-X plus cluster (Figure 6). The type of neurotoxin regulatory gene (*cntR*) present, therefore, is entirely in accordance with the type of neurotoxin gene cluster, but not with the type of neurotoxin gene.

### Neurotoxin cluster arrangement – Dual toxin gene strains

Twenty-two strains tested in the present study possessed two distinct neurotoxin genes. Fourteen of the dual gene toxin strains possessed a type A1 and type B neurotoxin gene. Two of these strains (CDC 657 and CDC 588) form both neurotoxins, albeit in different proportions, while the other 12 strains appear to form only type A neurotoxin (Figure 6). All these 14 strains gave an identical response in that they possessed complete ha plus/orf-X minus and ha minus/orf-X plus clusters. The microarray data cannot distinguish between dual toxin gene strains which carry a type A1 toxin gene in a ha minus/orf-X plus cluster, and a type B gene in a ha plus/orf-X minus cluster or vice-versa. However, as all type B neurotoxin genes have been associated with a ha plus/orf-X minus cluster (Figure 6; [9,12]), the simplest explanation is that the dual toxin gene strains are in the former arrangement. This has been reported in strains NCTC 2916 and CDC 657 [9,36,41] and from a preliminary analysis of strains NCTC 11199, MDa10, 667 and CDC 588 [18,41]. The four strains that formed both type B and type F toxin showed a similar hybridisation profile to the A1(B) strains except that they possessed a type F toxin gene rather than a type A toxin gene. Again both the full ha plus/orf-X minus and ha minus/orf-X plus clusters are present (Figure 6). It is likely that these strains possess a type F gene in a ha minus/orf-X plus cluster, plus a type B gene in a ha plus/orf-X minus cluster. This hypothesis is supported by (i) this is the pattern found in strains forming either type B or type F toxin, (ii) such an arrangement was indicated by a preliminary analysis of strain CDC 3281 [42], and (iii) was reported for a recently sequenced unnamed Bf strain [GenBank: NZ_ABDP00000000].

### Identification of a new toxin sub-type

**Figure 7:**
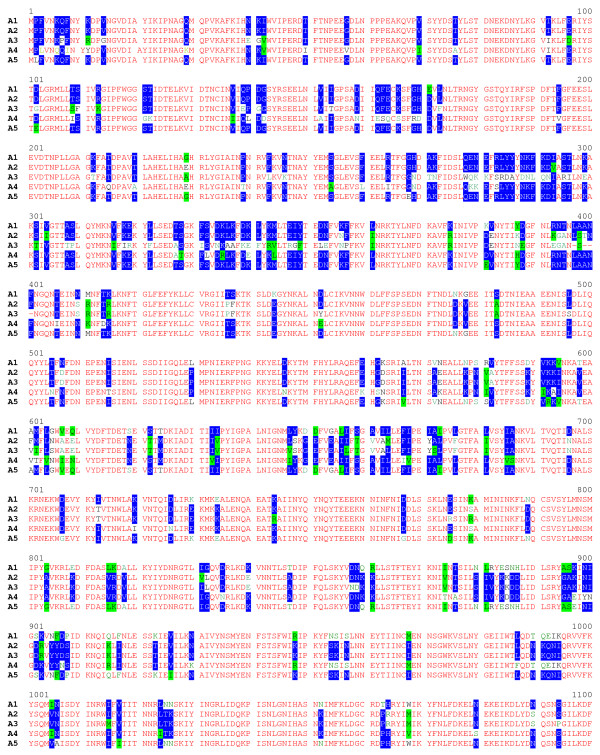
**Amino acid sequence alignment of proteolytic *C. botulinum *type A neurotoxin subtypes (part 1)**. Identical residues are in red; conservative differences are in white with blue background; blocks of similar residues are in black with green highlights; weakly similar residues are in green and non-similar residues are in black. Predicted amino acid sequences derive from published (GenBank) DNA sequence of: A1, ATCC 3502; A2, Kyoto F; A3, NCTC 2012; A4, CDC 657; A5, H0 4402 065 (this work).

**Figure 8:**
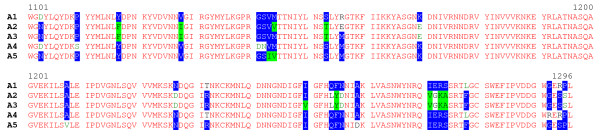
**Amino acid sequence alignment of proteolytic *C. botulinum *type A neurotoxin subtypes (part 2)**. Identical residues are in red; conservative differences are in white with blue background; blocks of similar residues are in black with green highlights; weakly similar residues are in green and non-similar residues are in black. Predicted amino acid sequences derive from published (GenBank) DNA sequence of: A1, ATCC 3502; A2, Kyoto F; A3, NCTC 2012; A4, CDC 657; A5, H0 4402 065 (this work).

**Figure 9:**
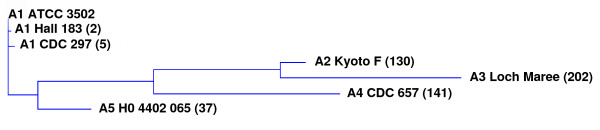
**Relatedness of *C. botulinum *type A neurotoxins**. The dendrogram was generated with the AlignX (ClustalW) programme of the Vector NTI Advance 10 (Invitrogen) software package, using data presented in Table 2 and Figures 7 and 8. Figures in brackets refer to the number of amino acid residues different to those of the A1 neurotoxin of ATCC 3502.

The present study included four strains of proteolytic *C. botulinum *(H0 4244 0055, H0 4402 065, H04464 107, H0 4068 0341) that formed type A neurotoxin, and had been isolated from patients presenting with wound botulism in different regions of the UK in 2004. Whole genome analysis revealed that these strains formed a sub-group within clade 3, distinct from other type A strains. Since the majority of strains forming type A neurotoxin clustered together within clades 4 or 7, this suggested the possibility that they might represent an evolutionary distinct group which could be sufficiently diverged to also produce a novel neurotoxin sub-type (Figure 1). From the DNA sequence of the entire *cntA *coding region, a translation product could be predicted that comprised 1297 amino acid residues of a type A neurotoxin gene. The *cntA*/A gene sequences from all four strains were identical suggesting that these strains may derive from a common source. Comparison with published examples of neurotoxin A sub-types revealed that the wound botulism-derived *cntA*/A genes were distinct from toxin sub-types A1 – A4 (Figures 7, 8 and 9; Table 2). Subtypes of *cntA *are defined by a minimum of 2.6% difference between amino acid sequences [7,10]. The closest relative of the wound botulism-derived *cntA*/A gene is the *cntA*/A1 gene (Table 2), and the new DNA sequence predicts a 2.9% difference (37 amino acid residues) between the wound botulism-derived *cntA*/A genes and the *cntA*/A1 genes, the latter tending to share approximately 99.8% identity between themselves (see Figures 7 and 8 for an alignment of amino acid sequence of all five sub-types). On this basis these wound botulism-derived *cntA*/A genes define a new sub-type, and should be termed *cntA*/A5. Furthermore, the four type A5(B) strains represented the only 'non-A1' neurotoxin-forming strains that possessed a type A neurotoxin gene in a ha plus/orf-X minus cluster (Figure 6). Interestingly all four type A5 strains gave a positive signal with the C-terminal type B probe. Following a combination of DNA sequencing and PCR analysis, the presence of a near complete type B neurotoxin gene with the 5' end (i.e. N-terminus of protein) either absent or diverged from previously published examples was detected (data not shown). As such, these strains also represent the first published examples of type A(B) strains that lack the ha minus/orf-X plus cluster for neurotoxin genes. Since active type B toxin was not detected in the mouse test, they are designated as type A5(B). Wound botulism cases in the UK are associated with heroin abuse [43], and it is likely that the source of these strains of proteolytic *C. botulinum *is the same as the heroin, which comes from Afghanistan [44]. This may indicate that previously unknown botulinum neurotoxin types are present in this part of Asia; the majority of published botulinum neurotoxin gene sequences are from strains originating in Europe or North America.

**Table 2:** Amino acid homology of neurotoxin A subtypes.

**Strain**	**ATCC 3502**	**Hall 183**	**CDC 297**	**Kyoto F**	**NCTC 2012**	**CDC 657**	**H0 4402 065**
**ATCC 3502**	**100.0 **(0)						
**Hall 183**	**99.8 **(2)	**100.0 **(0)					
**CDC 297**	**99.6 **(5)	**99.6 **(5)	**100.0 **(0)				
**Kyoto F**	**90.0 **(130)	**89.8 **(132)	**89.7 **(134)	**100.0 **(0)			
**NCTC 2012**	**84.4 **(202)	**84.3 **(204)	**84.2 **(205)	**93.0 **(91)	**100.0 **(0)		
**CDC 657**	**89.1 **(141)	**89.0 **(143)	**89.0 **(142)	**88.1 **(154)	**84.1 **(206)	**100.0 **(0)	
**H0 4402 065**	**97.1 **(37)	**97.1 **(38)	**96.9 **(40)	**90.3 **(125)	**84.9 **(196)	**87.3 **(165)	**100.0 **(0)

Pair-wise comparison of 1296 amino acid residue type A neurotoxins. ATCC 3502, Hall 183, CDC 297, type A1; Kyoto F, type A2; NCTC 2012, type A3; CDC 657, type A4; H0 4402 065, type A5. Bold figures: percent homology; figures in brackets: number of different residues. Only unique data are shown; A1 neurotoxins identical to that of strain ATCC 3502 include those of strains NCTC 3805 (62A), NCTC 7272 (ATCC 19397), and ATCC 25763. The A1 neurotoxin of Hall 183 is identical to that of the A1 (B) strain NCTC 2916. The Kyoto F A2 neurotoxin is identical to that of the FRI-Honey strain (all sequence data from GenBank). All type A5 neurotoxins described in this study are identical.

The amino acid residue differences that distinguish the A5 sub-type from the four other type A sub-types are scattered throughout its length (Figures 7 and 8; [7-9]). The N-terminal eight amino acid residues are involved in binding to the neuronal cell plasma membrane [45]. Significantly the A5 neurotoxin has a leucine at position 2, in contrast to the usual proline, an amino acid known to cause marked conformational changes in peptide secondary structure. The C terminus of the light chain, especially residues 398–448 is important for solubility, stability and catalysis [46], but only one residue (E444) close to the protease nicking site differs in this region. Similarly, of the heavy chain residues that are proposed to build the lactose and sialyllactose-binding pockets needed for ganglioside binding [47], only L1278 has been changed (to an F). It is tempting to predict from this *in silico *study that the gene product of *cntA*/A5 will share a similar toxicity to that of *cntA*/A1, although the fact that at least three residues known to be functionally important are different may have important implications.

### Relationship to *C. sporogenes*

The type B toxin producing strain 2345 had a weak signal for all four neurotoxin-associated probes (Figure 6), and also groups together with the three *C. sporogenes *strains in the whole genome analysis (Figure 1). These observations support a hypothesis that strain 2345 may represent a strain of *C. sporogenes *which has recently acquired part of the (or a diverged but intact) neurotoxin gene cluster in the recent evolutionary past. Interestingly, two of the three *C. sporogenes *strains, which were expected to be completely negative for all neurotoxin-associated probes, gave a weak signal to the *cntR/A1 *probe (Figure 6). This could be due to the presence of a distantly related or partial *cntR *gene, implying that these *C. sporogenes *strains may either represent a descendent of *C. botulinum *that have lost most of their ha plus/orf-X minus neurotoxin gene cluster, or may have acquired a neurotoxin gene cluster by horizontal gene transfer (as postulated for strain 2345), but then subsequently lost most of it. Although not the same strain as the three used in this work, BLAST searches of the predicted peptides of the (unfinished) *C. sporogenes *strain ATCC 15579 genome sequence showed that of several proteolytic *C. botulinum *examples of this gene (and one non-proteolytic *C. botulinum *example, that of strain Eklund 17B), CntR of ATCC 3502 gave the highest percentage identity (48%) over the longest unbroken stretch of peptide sequence. Whereas this tends to support the microarray data, the stretch of sequence was only 27 amino acid residues in length, so genome sequence analysis of more *C. sporogenes *strains would be needed to further investigate this interesting observation.

### Evolution of neurotoxin genes in relationship to the genome

It is evident that in strains of proteolytic *C. botulinum*, the distribution of neurotoxin genes and neurotoxin cluster type are not consistent with the whole genome analysis (Figure 6, Figure 1). This is consistent with previous reports by other genomic indexing methods (e.g. 16S *rrn*, PFGE, AFLP, MLST [2,8,32,33]). The evolutionary patterns of neurotoxin and associated genes within the neurotoxin cluster are also incompatible, and are likely to have arisen from several distinct recombination events. For example, the present study has confirmed earlier reports [18,38] that in type A1 strains, the neurotoxin genes may be located in a ha plus/orf-X minus cluster or in a ha minus/orf-X plus cluster. It was also found that the *cntR *gene correlated with neurotoxin cluster type, rather than neurotoxin gene. Previous reports have identified that NTNH-encoding genes also correlated with neurotoxin cluster type rather than neurotoxin gene, and that the middle of the NTNH gene may be a hot-spot for recombination events within the neurotoxin cluster [2,9,13]. Putative insertion sequence (IS) elements located close to the neurotoxin cluster and the localisation of neurotoxin genes on large plasmids may also have played a role in mobilisation and gene transfer of neurotoxin and associated genes [9,39].

### Flagellar glycosylation island (FGI)

Microarray analysis of the CDSs within the FGI separated strains into six divisions. Divisions 1 and 2 had similar profiles, with division 1 missing some CDSs contained within FGI-II. Divisions 3–6 were all missing large sections of FGI-II, with division 5 also missing CDSs within FGI-I (Figure 2). The structure of these divisions indicates that, as seen for the neurotoxin cluster genes, the evolution of the FGI may have occurred independently of the remainder of the genetic complement, with most divisions containing strains of more than one toxin type and from more than one clade. Divisions 1–3 each contained strains of at least four toxin types (or sub-types) that belonged to three or more clades, while division 4 contained only type B strains from clade 3, and division 5 comprised only the strains in clade 9 (type B and *C. sporogenes *strains). Division 6 contained just one strain, 17A. The genetic variation highlighted by these divisions (Figure 2) forms the basis for a typing method for proteolytic *C. botulinum *[29].

The genetic repertoire of the FGI indicated by the microarray analysis suggested that the glycan biosynthetic capacity of these *C. botulinum *strains may vary (Figure 2). Indeed, it has been shown that strains differ in their FlaA glycan structure, and it is proposed that FGI-II is involved in this process [29,31]. Flagellin proteins were isolated from representative strains in each division to determine the nature of the glycan produced and its correlation with FGI microarray profiles. Top down MS analysis of intact flagellin protein revealed diversity in glycan composition amongst isolates [30,31]; the masses of which are shown in Figure 2. Flagellin from the majority of strains in division 1 harboured a glycan oxonium ion at m/z 259. Further characterisation of this glycan by MS/MS showed fragmentation ions at m/z 200.1, 182.1, 158.1, 154.1, 126.1 and 112.1, characteristic of a di-N-acetylhexuronic acid, previously identified as part of a trisaccharide modification on *Methanococcus voltae *flagellin [48]. Many of the flagellins of division 2 strains carried a glycan oxonium ion at m/z 301. The MS/MS spectrum of this ion also shared fragmentation ions with a di-N-acetylhexuronic acid, but with the increased mass likely to correspond to the addition of a third acetyl group (data not shown). A glycan oxonium ion at m/z 418 was detected as the FlaA modification on all division 3 strains examined. This FlaA glycan has been fully characterized in strain FE9909ACS as a novel legionaminic acid derivative, Leg5Ac7NMeGlu [31]. Strains 920A276 and CDC 15044 from division 4 possessed flagellins with glycan oxonium ions at m/z 417 and 431, which shared glycan oxonium ion fragmentation patterns typical of nonulosonic acid sugars (data not shown). The flagellin of the division 5 strain FE0507BLP had a glycan oxonium ion at m/z 317, which had the characteristic MS/MS fragmentation pattern of the nonulosonic acid sugars pseudaminic and legionaminic acid (data not shown). These sugars have been structurally characterised from the flagellins of *Campylobacter jejuni *[49], *Campylobacter coli *[50] and *Helicobacter pylori *[51]. Taken together, these observations show that differences in the FGI microarray profiles may be reflected in the mass of glycan oxonium ions that modify the flagellin. Interestingly, top down mass spectrometry analysis of FlaA from strain 17A did not produce any marker ions characteristic of glycan, although the mass of the protein is greater than could be predicted from its DNA sequence. This indicates that it too is probably post-translationally modified [29]. In this case a 'bottom up' mass spectrometry analysis of flagellar tryptic peptides may be required to identify the glycan moiety.

A representative of division 3, which appears to produce the novel legionaminic acid derivative, Leg5Ac7NMeGlu, is the type F strain Langeland, the genome of which has been recently sequenced. Comparison of FGI sequences of both Langeland and ATCC 3502 showed that CDSs of FGI-I shared at least 80% identity, while FGI-II was highly divergent and was 30 kb smaller in strain Langeland (Figure 2). Homologues to the biosynthetic genes for legionaminic acid synthesis in *Campylobacter coli *have been identified in the FGI-II region of the Langeland genome [31]. The definitive confirmation that CDSs in FGI-II are responsible for biosynthesis of the glycan found on *C. botulinum *strain Langeland FlaA, however, awaits further genetic analysis.

Previously, nonulosonic acid sugars such as legionaminic acid and pseudaminic acid have been identified as the post-translational modification of flagellins in the Gram-negative gastrointestinal pathogens, *Campylobacter *and *Helicobacter *[52]. In these bacteria, the glycosylation of the flagellin is essential for filament assembly and glycan modifications have been shown to play a role in pathogenesis [53,54]. The presence of nonulosonic acid sugars in numerous strains of *C. botulinum *may have an important bearing on its ability to establish a gut infection and thereby cause infant or adult intestinal botulism. Although the present study has not correlated the distribution of specific flagellin glycan modifications with the type of botulism caused, this property may enable a strain to bring about infant/adult intestinal botulism at a lower infectious dose than that of strains lacking these flagellin modifications. A comparative genomic analysis of *Campylobacter jejuni *identified distinct distributions of flagellar glycosylation genes (*cj1321–cj1326*) that were present only in strains associated with colonisation of livestock [53]. The hypothesis was developed that the type of flagellar glycosylation genes in *Campylobacter jejuni *strains conferred a survival advantage to these strains within livestock, offering a possible explanation for the host specificity of some *Campylobacter jejuni *strains. It remains to be established whether diversity in flagellar glycan biosynthetic capacity in *C. botulinum *is similarly related to host specificity and the colonization ability of isolates.

## Conclusion

The most important aspects of the biology and evolution of proteolytic *C. botulinum *have been highlighted by this study, particularly in relation to the neurotoxin and its associated cluster and the FGI. The close relationship with *C. sporogenes*, and very distant relationship with non-proteolytic *C. botulinum *have been confirmed. Proteolytic *C. botulinum *and non-proteolytic *C. botulinum *are phylogenetically distinct organisms that coincidentally share type B and type F neurotoxin genes. These genes are of such sequence similarity as to obviously share a recent common ancestor, and appear therefore to have crossed the species barrier. Intriguingly, type A and type E neurotoxin genes seem to be mutually exclusive and are each restricted to just one of these species. The genome of proteolytic *C. botulinum *appears to be relatively stable, and strains sequenced to date display a high degree of synteny (data not shown). There are, however, variable regions, and we have presented evidence for the independent horizontal transfer of genes encoding the neurotoxin cluster and FGI, compared to the remainder of the genetic complement. Transfer of neurotoxin and associated genes may be associated with a hot-spot for recombination within the NTNH, closely associated IS elements, and plasmids. Further investigations of unexpected toxin or FGI types within clades may be particularly interesting, and reveal more about the acquisition or loss of genetic material. For example, while most type A1 strains grouped together according to whole genome and FGI analysis, four appeared to be distinct. Two of these type A1 strains, FE9604A and MUL0109ASA were closely related to each other, with the toxin gene in a ha minus/orf-X plus cluster. Strains 17A and 96A were both ha plus/orf-X minus strains, but appear to be different to each other and all the other type A1 strains by whole genome and FGI analysis. Interestingly, strain 96A was also well separated from other type A1 strains by PFGE [32]. The sequencing of further strains (which is rapidly becoming affordable for most laboratories) is a particularly attractive way forward, as unlike microarray analysis, which can only highlight CDSs that are present or absent in a test strain, it will provide information not only on what has been inserted or lost, but where on the genome this has taken place. Indeed the genomes of several of the strains used in this study have recently been sequenced, and those with slightly larger genomes typically also carry approximately 300–600 novel genes with respect to the ATCC 3502 strain used as a reference in this work (data not shown).

A number of typing tools have been used for the molecular characterisation of proteolytic *C. botulinum*. Some (e.g. ribotyping, 16S *rrn *sequencing) can be used to identify the organism, but are not particularly effective at discriminating between strains [8,55]. Others (e.g. PFGE, MLST, AFLP, VNTR, *fla *sequencing, DNA microarrays) are able to discriminate between strains [8,14,29,32-34]. The present study and previous work [14] have demonstrated that comparative genomic indexing using a DNA microarray based on the genome sequence of ATCC 3502 is an effective tool to discriminate strains of proteolytic *C. botulinum*. Advantages of microarrays are that they can infer evolutionary relationships better than single/multi-locus methods and additionally provide valuable information on the genome content of tested strains, thereby providing an insight into the biology and evolution of the organism. The present microarray is suitable for the forensic analysis of strains of proteolytic *C. botulinum*, including investigations of bioterrorism associated events. A second generation DNA microarray could be developed for this purpose based on the variable regions identified between a number of sequenced strains, and utilise printed rather than spotted microarrays.

## Authors' contributions

MWP and JWA obtained funds for, initiated and conceived this study. ATC, CJP and DRM carried out the comparative genomic indexing work. ATC carried out the analysis with a contribution from MA. CJP, SMT and SML performed the FGI analysis. ATC and MWP coordinated the writing of the manuscript, with all authors providing critical feedback. All authors read and approved the final manuscript.
